# Evaluation of the Predictive Role of Blood-Based Biomarkers in the Context of Suspicious Prostate MRI in Patients Undergoing Prostate Biopsy

**DOI:** 10.3390/jpm11111231

**Published:** 2021-11-19

**Authors:** Pawel Rajwa, Nicolai A. Huebner, Dadjar I. Hostermann, Nico C. Grossmann, Victor M. Schuettfort, Stephan Korn, Fahad Quhal, Frederik König, Hadi Mostafaei, Ekaterina Laukhtina, Keiichiro Mori, Reza Sari Motlagh, Takafumi Yanagisawa, Abdulmajeed Aydh, Piotr Bryniarski, Benjamin Pradere, Andrzej Paradysz, Pascal A. Baltzer, Bernhard Grubmüller, Shahrokh F. Shariat

**Affiliations:** 1 Department of Urology, Medical University of Silesia, 41-800 Zabrze, Poland; piotr.bryniarski@hotmail.com (P.B.); parady@poczta.onet.pl (A.P.); 2 Department of Urology, Medical University of Vienna, 1090 Vienna, Austria; nicolai.huebner@meduniwien.ac.at (N.A.H.); dadjar.hostermann@googlemail.com (D.I.H.); nico.grossmann@gmail.com (N.C.G.); victor.schuettfort@gmail.com (V.M.S.); stephan.korn@meduniwien.ac.at (S.K.); fahad.quhal@meduniwien.ac.at (F.Q.); frederik.koenig1@gmail.com (F.K.); Hadimosta@gmail.com (H.M.); katyalaukhtina@gmail.com (E.L.); morikeiichiro29@gmail.com (K.M.); motlagh.reza.dr@gmail.com (R.S.M.); t.yanagisawa.jikei@gmail.com (T.Y.); mageed18@hotmail.com (A.A.); benjaminpradere@gmail.com (B.P.); bernhard.grubmueller@meduniwien.ac.at (B.G.); shahrokh.shariat@meduniwien.ac.at (S.F.S.); 3 Working Group for Diagnostic Imaging in Urology (ABDU), Austrian Association of Urology (ÖGU), 1090 Vienna, Austria; 4 Department of Urology, Luzerner Kantonsspital, 6000 Lucerne, Switzerland; 5 Department of Urology, University Hospital Zurich, 8091 Zurich, Switzerland; 6 Department of Urology, University Medical Center Hamburg-Eppendorf, 20251 Hamburg, Germany; 7 Department of Urology, King Fahad Specialist Hospital, Dammam 32253, Saudi Arabia; 8 Research Center for Evidence Based Medicine, Tabriz University of Medical Sciences, Tabriz 51666-15731, Iran; 9 Institute for Urology and Reproductive Health, Sechenov University, 19435 Moscow, Russia; 10 Department of Urology, The Jikei University School of Medicine, Tokyo 105-8461, Japan; 11 Men’s Health and Reproductive Health Research Center, Shahid Beheshti University of Medical Sciences, Tehran 19857-17443, Iran; 12 Department of Biomedical Imaging and Image-Guided Therapy, Medical University of Vienna, 1090 Vienna, Austria; pascal.baltzer@meduniwien.ac.at; 13 Karl Landsteiner Institute of Urology and Andrology, 1010 Vienna, Austria; 14 Department of Urology, Weill Cornell Medical College, New York, NY 10065, USA; 15 Department of Urology, University of Texas Southwestern, Dallas, TX 75390, USA; 16 Department of Urology, Second Faculty of Medicine, Charles University, 150 06 Prague, Czech Republic; 17 Department of Special Surgery, Jordan University Hospital, The University of Jordan, Amman 11942, Jordan

**Keywords:** MRI, biopsy, dNLR, NLR, PNI, prostate cancer

## Abstract

The aim of this study was to assess the predictive value of pre-biopsy blood-based markers in patients undergoing a fusion biopsy for suspicious prostate magnetic resonance imaging (MRI). We identified 365 consecutive patients who underwent MRI-targeted and systematic prostate biopsy for an MRI scored Prostate Imaging–Reporting and Data System Version (PI-RADS) ≥ 3. We evaluated the neutrophil/lymphocyte ratio (NLR), derived neutrophil/lymphocyte ratio (dNLR), platelet/lymphocyte ratio (PLR), systemic immune inflammation index (SII), lymphocyte/monocyte ratio (LMR,) de Ritis ratio, modified Glasgow Prognostic Score (mGPS), and prognostic nutrition index (PNI). Uni- and multivariable logistic models were used to analyze the association of the biomarkers with biopsy findings. The clinical benefits of biomarkers implemented in clinical decision-making were assessed using decision curve analysis (DCA). In total, 69% and 58% of patients were diagnosed with any prostate cancer and Gleason Grade (GG) ≥ 2, respectively. On multivariable analysis, only high dNLR (odds ratio (OR) 2.61, 95% confidence interval (CI) 1.23–5.56, *p* = 0.02) and low PNI (OR 0.48, 95% CI 0.26–0.88, *p* = 0.02) remained independent predictors for GG ≥ 2. The logistic regression models with biomarkers reached AUCs of 0.824–0.849 for GG ≥ 2. The addition of dNLR and PNI did not enhance the net benefit of a standard clinical model. Finally, we created the nomogram that may help guide biopsy avoidance in patients with suspicious MRI. In patients with PI-RADS ≥ 3 lesions undergoing MRI-targeted and systematic biopsy, a high dNLR and low PNI were associated with unfavorable biopsy outcomes. Pre-biopsy blood-based biomarkers did not, however, significantly improve the discriminatory power and failed to add a clinical benefit beyond standard clinical factors.

## 1. Introduction

Over the last decade, magnetic resonance imaging (MRI) has become an essential tool for the diagnosis and management of prostate cancer (PCa) [1,2]. Indeed, prostate MRI allows visualization and assessment of the extent of suspicious lesions, in addition to guiding targeted biopsies [3,4,5]. This strategy has led to a higher likelihood of detecting clinically significant PCa (International Society of Urological Pathology (ISUP) Gleason Grade (GG) ≥ 2) compared to the standard systematic biopsy [1,3,4,5]. Several prostate MRI standardized reporting schemes have been developed, with Prostate Imaging–Reporting and Data System version 2 (PI-RADS v2) being the most frequently employed scheme [6]. In the clinical setting, major urological guidelines recommend performing MRI-targeted and systematic biopsy in patients with a PI-RADS ≥ 3 lesion [1,6]. Although the PI-RADS classification limits the intrareader variability and allows, with approximately 80% accuracy, to exclude clinically significant PCa, the overall predictive value remains low to moderate. A recent meta-analysis found a pooled significant PCa detection rate of 17% for PI-RADS 3, 46% for PI-RADS 4, and 75% for PI-RADS 5 [7]. These results suggest that 25–83% of males with suspicious prostate MRI undergo unnecessary biopsies and could benefit from the enhanced risk stratification to avoid an unpleasant, invasive procedure with significant associated risk. 

Regarding the necessity of a biopsy, clinical data and biomarkers such as PSA and PSA density (PSAD) have been shown to help guide decision-making in patients with suspected prostate MRI [1,8,9]. Few genomic assays have shown improved detection rates; however, their use is limited owing to the high cost and limited availability [1,10,11]. Several blood-based biomarkers, which combine immune cell counts, have been proposed to have potential diagnostic, predictive, and prognostic values of different disease states [12,13]. These biomarkers represent a systemic inflammatory burden, as immune cells interplay with the cancer-related environment. For instance, neutrophils, platelets, or lymphocytes play a vital role in tumor development, progression, and dissemination [12,13,14]. Most studies analyzing the diagnostic utility of blood-based biomarkers focused on single biomarkers and were tested before the MRI era.

Our primary goal was to analyze the predictive value of multiple biomarkers, including the neutrophil/lymphocyte ratio (NLR), derived neutrophil/lymphocyte ratio (dNLR), platelet/lymphocyte ratio (PLR), systemic immune inflammation index (SII), lymphocyte/monocyte ratio (LMR,) de Ritis ratio, modified Glasgow Prognostic Score (mGPS), and prognostic nutrition index (PNI) in patients with suspicious prostate MRI lesions undergoing MRI-targeted and systematic prostate biopsy. The secondary aim was to determine if the biomarkers can help avoid biopsies in patients with suspicious MRI lesions. 

## 2. Materials and Methods

We retrospectively reviewed our institutional MRI biopsy database and identified patients with prostate MRI PI-RADS ≥ 3 lesions who underwent targeted and systematic diagnostic biopsy between 2017 and 2019. The local institutional review board approved this study (EC no. 2209/2019)**.** In general, patients underwent prostate MRI in the case of suspicious digital rectal examination (DRE) or PSA > 4 ng/mL. No patient underwent any type of PCa therapy, prostate surgery, or oral 5α-reductase inhibitor before the biopsy. All prostate MRIs were performed using a body coil on a 3 Tesla MRI according to the European Society of Urogenital Radiology (ESUR) PI-RADS recommendations. No endorectal coil was used. All patients underwent MRI with T2-weighted imaging (T2WI), diffusion-weighted imaging (DWI), and dynamic contrast-enhanced (DCE) sequences. Images were assessed in line with PI-RADS v2 classification by radiologists experienced in prostate MRI [6]. Patients underwent transrectal MRI-ultrasound fusion biopsy using the UroNav System (Invivo Corporation, PHILIPS©, 3545 SW47th Avenue, Gainesville, Florida 32608 USA), with targeted cores sampled from all MRI regions of interest under local anesthesia. The obtained biopsy specimens were examined centrally using the ISUP grade group (GG) classification in line with ISUP 2014 recommendations [15]. 

### 2.1. Biomarkers

Biomarkers data were retrieved from in-house pre-biopsy complete blood count. We calculated the ratios using absolute counts of cells and inflammatory indices as previously reported [12,16,17]—NLR: neutrophils/lymphocyte; dNLR: neutrophils/leukocytes—neutrophils; PLR: platelets/lymphocytes; LMR: lymphocytes/monocytes; SII: neutrophils x platelets/lymphocytes; de Ritis aspartate aminotransferase (AST)/alanine aminotransferase (ALT); mGPS as previously described in detail [18]; PNI 10× serum albumin (g/dL) + 0.005 × total lymphocyte count (per mm^3^). Pre-biopsy optimal cut-offs were determined by receiver operating characteristics (ROC) curve analysis using the Youden index for GG ≥2 prediction. In summary, the Youden index provides the optimal cut-off from a continuous variable by showing the score that offers the best tradeoff between sensitivity and specificity. 

### 2.2. Statistical Analyses 

Associations between biomarkers and patients’ clinicopathologic features were evaluated using the Kruskal–Wallis rank sum test for continuous variables and chi-square test of independence or Fisher’s exact test for categorical variables, as appropriate. Univariable and multivariable logistic regression analyses tested the association of biomarkers with any PCa and GG ≥ 2. We tested the models’ predictive accuracy using receiver operating characteristics (ROC) curves and calculated the derived area under the curve (AUC). The AUCs were statistically compared using DeLong’s test. Based on multivariable logistic regression models, nomograms were created to guide clinical decision-making. Decision curve analysis was used to analyze the clinical net benefit of biomarkers. Analyses were performed using R Version 4.0 (R Foundation for Statistical Computing, Vienna, Austria, 2020).

## 3. Results

We identified 365 patients with suspicious MRI lesions, out of whom 324 had available in-house pre-operative complete blood counts, which allowed us to calculate biomarkers’ values. The overall cohort characteristics and those stratified by PI-RADS scores and cancer status are presented in Table 1. Of all suspicious MRIs, 11% were scored as PI-RADS 3, 52% as PI-RADS 4, and 37% as PI-RADS 5. The median PSA and PSAD values, age, DRE status, and the PCa detection rate differed between these PI-RADS categories (all *p* < 0.01). There were no differences in terms of biomarkers levels. GG ≥ 2 disease was diagnosed in 14% of PI-RADS 3 lesions, 52% in PI-RADS 4 lesions, and 79% of PI-RADS 5 lesions; there were also significant differences for core positivity between each PI-RADS category. Moreover, patients with GG ≥ 2 were older (*p* < 0.001) and had higher values of PSAD (*p* < 0.001), NLR (*p* = 0.045), dNLR (*p* = 0.061), de Ritis ratio (p=0.045), and lower values of PNI (*p* = 0.002). A similar number of biopsy cores were sampled in patients with benign or GG1 and GG ≥ 2. The optimal marker cut-offs, determined by ROC curve analysis for GG ≥ 2, were: NLR ≥ 2.75, dNLR ≥ 2.06, PLR ≥ 133.5, LMR < 2.07, de Ritis ratio ≥ 1.11, SII ≥ 272.6, and PNI < 52.8 (Supplementary Table S1). 

On univariable logistic regression analyses (Supplementary Table S2) along with standard clinical factors, NLR (OR 2.39, 95% CI 1.41–4.05, *p* = 0.001), dNLR (OR 3.67, 95% CI 1.91–7.07, *p* < 0.001), LMR (OR 0.35, 95% CI 0.16–0.75, *p* = 0.007), and PNI (OR 0.39, 95% CI 0.24–0.65, *p* < 0.001) were associated with GG ≥ 2 prediction. 

On multivariable analyses (Table 2), adjusted for the effects of age, PSAD, DRE, and PI-RADS category, a high dNLR predicted both PCa (OR 2.63, 95% CI 1.09–6.35 *p* = 0.032) and GG ≥ 2 (OR 2.61, 95% CI 1.23–5.56, *p* = 0.017). In the same multivariable model, PNI was also an independent predictor for both PCa (OR 0.39, 95% CI 0.20–0.78, *p* = 0.008) and GG ≥ 2 (OR 0.48, 95% CI 0.26–0.88, *p* = 0.018). For NLR, the results for GG ≥ 2 prediction were close (OR for GG ≥ 2: 1.82, 95% 0.98–3.38, *p* = 0.057). 

**Figure 1:**
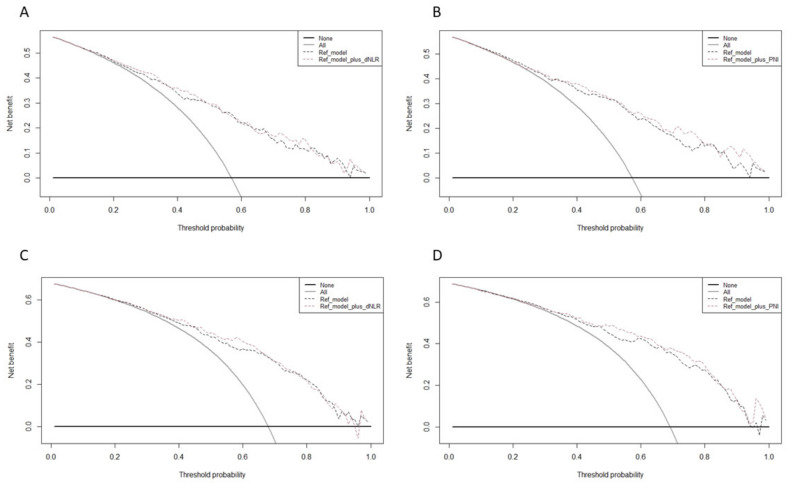
Decision curve analysis (DCA) for the net benefit of the pre-biopsy biomarkers based on the reference model for the prediction of (**A**) dNLR for GG ≥ 2, (**B**) PNI for GG ≥ 2, (**C**) dNLR for PCA, and (**D**) PNI for PCa. Decision curve analysis evaluating the clinical impact of clinical pre-biopsy models (reference model) with the integration of biomarker (reference model plus **A**,**C**: dNLR; **B**,**D**: PNI) estimating (**A**,**B**) GG ≥ 2 and (**C**,**D**) PCA in patients with MRI score PI-RADS ≥ 3 undergoing MRI-targeted and systematic biopsy. The x axis is the threshold probability. The y axis measures the net benefit, which is calculated by adding the true positives and subtracting the false positives. The horizontal line representing the x axis assumes that no patients undergo biopsy, whereas the gray line assumes that all patients undergo biopsy at a specific threshold probability. The dashed black line represents the net benefit of the regression model that was fitted using established clinicopathological variables. The dashed red line represents the net benefit of the same regression models with biomarkers.

The logistic regression models, which comprised age, PSAD, DRE, PI-RADS categories, and dNLR or PNI achieved high accuracy for any PCa (AUCs: 0.831–0.865) and GG ≥ 2 (AUCs: 0.821–0.849). The addition of the biomarkers did not improve the clinical reference models by a statistically significant margin (*p* ≥ 0.05 for all). For prediction of any PCa and GG ≥ 2, the decision curve analysis showed that the clinical reference model with biomarkers offered a clinical net benefit relative to the all-patient biopsy approach at a threshold of 30–40% (Figure 1). However, the addition of dNLR and PNI did not (or only slightly) increase the net benefit relative to the reference model. 

**Figure 2:**
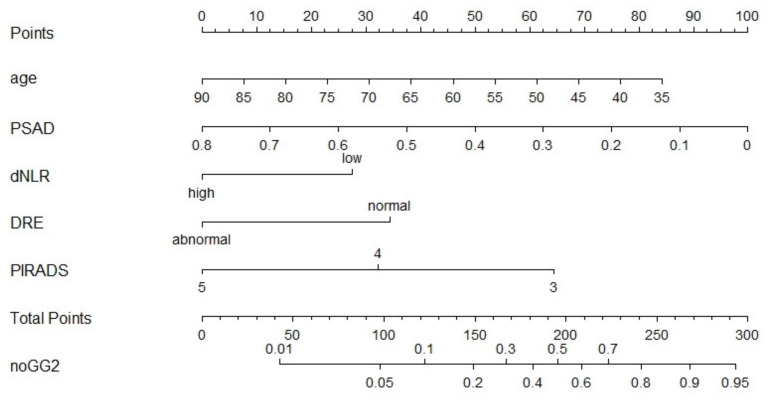
Pre-biopsy nomogram based on multivariable logistic regression model with dNLR predicting benign pathology or GG1 disease in patients with PI-RADS ≥ 3 lesions undergoing systematic and targeted biopsy. Instructions for physicians: Locate the patients status on the corresponding axis. Draw a straight line to determine how many points the patients should receive for each variable. Sum the points received and locate the number on the total points axis. Draw a line down from total points to the noGG2 axis.

**Figure 3:**
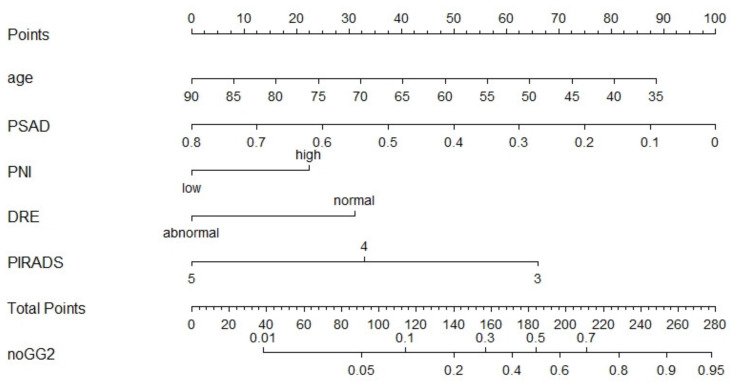
Pre-biopsy nomogram based on the multivariable logistic regression model with PNI predicting benign pathology or GG1 disease in patients with PI-RADS ≥ 3 lesions undergoing systematic and targeted biopsy. Instructions for physicians: Locate the patients status on the corresponding axis. Draw a straight line to determine how many points the patients should receive for each variable. Sum the points received and locate the number on the total points axis. Draw a line down from total points to the noGG2 axis.

The nomograms for benign histopathology or GG = 1 prediction, which comprised variables from the multivariable logistic regression models, demonstrated a range of predicted probabilities; however, PNI and dNLR did not contribute the highest number of risk points (Figure 2 and Figure 3). In the calibration plots (Supplementary Figures S1 and S2), the models showed near-optimal agreement between the models’ prediction and actual outcome observation; the goodness-of-fit tests were not significant (nomogram with dNLR: mean absolute error = 0.02; nomogram with PNI: mean absolute error = 0.016).

## 4. Discussion

The fully MRI-guided PCa diagnostic pathway is hampered by the poor positive predictive value of suspicious MRI, which leads to unnecessary biopsies and, despite the strong negative predictive value, a 10–20% risk of missing significant PCa. In the clinical setting, the present tools offer suboptimal accuracy to facilitate biopsy decision-making and patients’ selection in the case of a visible MRI lesion, too. Cheap, easily obtainable, and accurate biomarkers, which reflect the tumor-related inflammatory burden, could improve the current diagnostic strategies and minimize the risk of unnecessary biopsies and avoid missing significant PCa.

In this study, we demonstrate that pre-biopsy blood biomarkers and clinical parameters can help improve patients’ selection for prostate biopsy. Our results suggest that approximately 50% of patients with suspicious MRI do not have significant cancer on biopsy; the probability was highest for patients with PI-RADS 3 lesions (84%). Therefore, even patients with PI-RADS ≥ 3 lesions should undergo pre-biopsy risk stratification as it could spare unpleasant, unnecessary biopsies. Second, we found that other clinical factors enhanced with blood biomarkers achieve over 80% accuracy for detecting significant PCa prediction. Third, we found that out of all analyzed biomarkers, dNLR and PNI were the most valuable predictors and offered unique predictive information beyond PSA and clinical factors. Fourth, we constructed a nomogram as a ready-to-use tool that could help avoid biopsies in patients with suspicious prostate MRI undergoing targeted and systematic biopsy. 

In the era of precision medicine, with new imaging modalities and biomarkers, there is a persisting need to further improve pre-biopsy risk stratification. Negative and positive predictive values of MRI and single biomarkers highly depend on disease prevalence; therefore, combined strategies are needed [2,19,20]. Our model and the corresponding nomogram combining readily available biomarkers, including DRE, age, PI-RADS, and PSAD, revealed that even in patients with suspicious MRI, it is possible to include or exclude, with over 82% accuracy, the probability of significant PCa. This is particularly important as invasive biopsies are associated with pain, fever, and sepsis and may lead to hospitalization [21]. Furthermore, enhanced pre-biopsy risk stratification and MRI-guided diagnostic pathway can reduce health care costs and improve quality of life [22,23]. Recently, Deniffel et al. found that PSAD-based strategy in suspicious prostate MRI lesions (PI-RADS ≥ 3) can help reduce biopsies and outperforms MRI-based decision [24]. The analyzed and recalibrated models, which are well established, achieved AUCs of 0.79–0.84, which is comparable to ours [24]. However, in contrast to our cohort, their study included more patients with equivocal (PI-RADS 3) MRI lesions; PSA (median 7.8–7.9 ng/mL), age (median 65–67 years), and other clinical variables were similar to those of our cohort [24]. In the future, new tools such as artificial intelligence with radiomics may limit the MRI inter-reader variability and further enhance risk stratification in patients with suspicious MRI [19,20,25,26,27].

To our knowledge, we have presented the first study that specifically analyzed a panel of systemic inflammatory response biomarkers in PI-RADS ≥ 3 patients who underwent MRI-targeted and systematic biopsies. We found that patients with high dNLR were at two times higher risk of GG ≥ 2 and any PCa, irrespective of other confounders. Cancer can modulate polarization and excessive release of neutrophils, which are involved in tumor initiation and progression [28]. A decreased number of lymphocytes, which are the major component of dNLR denominator, is a poor prognosticator in cancer patients and is associated with excessive cancer expression of proapoptotic ligands [29,30]. Up to now, only a few studies have analyzed the predictive value of single or few blood biomarkers in the context of MRI-guided biopsies [31,32] and others in prostate MRI followed by radical prostatectomy [33]. In a study by Sun et al., who evaluated 335 men with both suspicious and non-suspicious MRIs, NLR (OR 2.37, 95% CI 1.38–4.06, *p* = 0.002), but not PLR or LMR, was an independent predictor of significant PCa [31]. Furthermore, the addition of NLR to the reference model (DRE, % free PSA, age) improved the AUC to 0.813, which was further enhanced by the incorporation of PI-RADS (AUC 0.873). Our model showed that dNLR, but not NLR (*p* = 0.06), was a significant predictor of any and GG ≥ 2 PCa. This is partly in line with the study of Pichler et al., who found that dNLR, but not NLR, independently predicted oncological outcomes in renal cell carcinoma patients [34]. Concordantly, in two prospective randomized trials, which included metastatic castration-resistant PCa (mCRPC) treated with chemotherapy, high (≥2) dNLR was prognostic for overall survival (hazard ratio 1.43, 95% CI 1.20–1.70, *p* < 0.001) [14]. 

No prior study evaluated the value of PNI in the context of prostate biopsy and only several in metastatic PCa [35,36]. In a study by Fan et al., PNI predicted response to abiraterone in patients with mCRPC [35]. In another study by Li et al., low PNI was associated with adverse oncologic outcomes in hormone-sensitive PCa treated with androgen-deprivation therapy [36]. In general, hypoalbuminemia is a poor prognostic factor in cancer patients and is associated with cancer-related inflammation and malnutrition [37]. High PNI, which combines albumins and lymphocytes, is a good prognostic factor in cancer patients [36,37]. We found that patients with high PNI were over 50% less likely to be diagnosed with GG≥2 on MRI-targeted and systematic biopsy, which suggests that with risk stratification using PNI and other clinical factors, some of the patients with suspicious MRI may forgo biopsy. Still, PCa is one of the most common causes of cancer-specific death [1]. Therefore, there is a need to improve the understanding of cancer development [38] and search for new PCa markers [39].

Our study has several limitations. First, there are limitations inherent to any retrospective data collection, especially concerning any potential selection bias. Second, some of the patients could suffer from chronic inflammatory conditions that could have influence markers levels; nevertheless, none of the patients suffered from acute inflammatory disease. Third, patients did not undergo pre-diagnosis and advanced imaging such as PSMA/PET-CT, and our staging was limited to DRE. Fourth, our nomograms lack external validation. Despite these limitations, we present the first study that comprehensively analyzes the role of multiple biomarkers in patients with suspicious prostate MRI undergoing MRI-targeted and systematic biopsy. Prospective studies are warranted to validate our results.

## 5. Conclusions

We found that despite suspicious prostate MRI, a meaningful number of patients would benefit from enhanced pre-biopsy risk stratification. In patients with PI-RADS ≥ 3 lesions undergoing MRI-targeted and systematic biopsy, high dNLR and low PNI, but not NLR, PLR, LMR, SII, and mGPS, were independent predictors of any PCa and/or GG ≥ 2. Nevertheless, these biomarkers did not improve the discriminatory ability of a reference model comprising DRE, PI-RADS category, PSAD, and age, which reached over 80% accuracy.

## Figures and Tables

**Table 1:** Consecutive patients with PI-RADS ≥ 3 lesions undergoing MRI.

	Overall	PI-RADS				GG ≥ 2		
Characteristic	*n* = 324	3, *n* = 37 (11%)	4, *n* = 168 (52%)	5, *n* = 119 (37%)	*p*-value	negative, *n* = 137 (42%)	positive, *n* = 187 (58%)	*p*-value
Age (years)	67 (60–73)	66 (58–72)	65 (59–72)	72 (63–75)	<0.001	65 (58–70)	69 (62–75)	<0.001
PSA (ng/mL)	7 (5–11)	7 (5–8)	7 (5–10)	9 (5–14)	0.001	6 (4–9)	8 (5–12)	<0.001
PSAD (ng/mL^2^)	0.17 (0.10–0.28)	0.14 (0.08–0.19)	0.16 (0.09–0.25)	0.23 (0.15–0.37)	<0.001	0.13 (0.08–0.19)	0.22 (0.14–0.35)	<0.001
DRE (cT ≥ 2) (%)	85 (26)	3 (8.1)	29 (17)	53 (45)	<0.001	15 (11)	70 (37)	<0.001
NLR	2.16 (1.66–2.88)	2.19 (1.58–2.67)	2.12 (1.57–2.75)	2.29 (1.72–3.15)	0.2	2.14 (1.57–2.61)	2.21 (1.69–3.08)	0.045
dNLR	1.51 (1.16–1.94)	1.61 (1.12–1.92)	1.46 (1.16–1.86)	1.60 (1.19–2.08)	0.3	1.47 (1.13–1.80)	1.54 (1.19–2.11)	0.061
PLR	124 (96–151)	117 (95–140)	125 (93–153)	128 (104–156)	0.3	120 (94–146)	128 (101–156)	0.2
LMR	3.17 (2.41–4.00)	3.12 (2.55–4.05)	3.20 (2.45–4.33)	3.00 (2.37–4.00)	0.13	3.20 (2.50–4.00)	3.14 (2.35–4.00)	0.3
SII	482 (359–651)	488 (341–633)	458 (358–637)	513 (366–674)	0.6	478 (366–629)	485 (350–674)	0.6
PNI *	54.5 (51.0–57.0)	54.7 (53.7–56.7)	54.8 (50.9–57.6)	53.8 (50.6–56.7)	0.3	54.8 (53.2– 57.2)	53.3 (50.5–56.6)	0.002
De Ritis ratio	0.96 (0.82–1.16)	0.95 (0.81–1.18)	0.93 (0.79–1.11)	1.00 (0.86–1.26)	0.084	0.94 (0.78–1.10)	1.00 (0.84–1.21)	0.045
mGPS **					>0.9			0.8
0 (%)	234 (87)	27 (87)	117 (86)	90 (88)		99 (86)	135 (88)	
1 (%)	34 (13)	4 (13)	18 (13)	12 (12)		16 (14)	18 (12)	
2 (%)	1 (0.4)	0 (0)	1 (0.7)	0 (0)		0 (0)	1 (0.6)	
No. of total cores	14 (12–16)	13 (13–14)	15 (12–16)	14 (10–16)	0.10	15 (12–16)	14 (12–16)	0.7
No. of targeted cores	4 (4–5)	4. (4–4)	4 (4–5)	4 (4–6.00)	0.4	4.00 (4.00–5.00)	4 (4–5)	0.8
No of systematic cores	1 (6–12)	10 (10– 12)	10 (8–12)	10 (6–12)	0.070	10 (8–12)	10 (6–12)	0.5
PCa (%)	222 (69)	9 (24)	107 (64)	106 (89)	<0.001	35 (26)	187 (100)	<0.001
GG≥2 (%)	187 (58)	5 (14)	88 (52)	94 (79)	<0.001	0	187 (100)	
>50% positive cores (%)	85 (26)	1 (2.7)	24 (14)	60 (50)	<0.001	3 (2.2)	82 (44)	<0.001

*n* (%); median (IQR); Kruskal–Wallis rank sum test; Pearson’s chi-squared test; Fisher’s exact test; * PNI (*n* = 278); ** mGPS (*n* = 269). Abbreviations: dNLR: derived neutrophil-lymphocyte ratio; DRE: digital rectal examination; GG: Gleason Grade; LMR: lymphocyte-monocyte ratio; *n*: number; mGPS: modified Glasgow prognostic score; NLR: neutrophil-lymphocyte ratio; PCA: prostate cancer; PI-RADS: Prostate Imaging-Reporting and Data System; PLR, platelet-lymphocyte ratio; PNI: prognostic nutrition index; PSA: prostate-specific antigen; PSAD: PSA density; SII: systemic immune-inflammation index.

**Table 2:** Consecutive patients with PI-RADS ≥ 3 lesions undergoing MRI-targeted and systematic biopsy stratified by PI-RADS and cancer status.

	GG ≥ 2 Prediction				PCa Prediction			
Biomarker	OR	95% CI	*p*-value	AUC (clinical model + biomarker)	OR	95% CI	*p*-value	AUC (clinical model + biomarker)
NLR (high vs. low) *	1.82	0.98–3.38	0.057	0.821	1.73	0.87–3.34	0.110	0.831
dNLR (high vs. low) *	2.61	1.23–5.56	0.017	0.824	2.63	1.09–6.35	0.032	0.834
LMR (high vs. low) *	0.62	0.25–1.54	0.302	0.820	0.35	0.11–1.13	0.079	0.828
PNI * (high vs. low)	0.48	0.26–0.88	0.018	0.840	0.39	0.20–0.78	0.008	0.865
				Clinical model ** AUC = 0.818				Clinical model ** AUC = 0.826

Abbreviations: AUC: area under curve; dNLR: derived neutrophil–lymphocyte ratio; DRE: digital rectal examination; GG: Gleason Grade; LMR: lymphocyte–monocyte ratio; *n*: number; NLR: neutrophil–lymphocyte ratio; PCa: prostate cancer; PI-RADS: Prostate Imaging–Reporting and Data System; PNI: prognostic nutrition index; PSA: prostate-specific antigen; PSAD: PSA density. * Corrected for PSAD, age, PI-RADS, DRE. ** Clinical model: PSAD, age, PI-RADS, DRE.

## Data Availability

The data presented in this study are available on request from the corresponding author. The data are not publicly available due to privacy and ethical restrictions.

## Supplementary Materials

The following are available online at https://www.mdpi.com/article/10.3390/jpm11111231/s1: Figure S1: Calibration plots of the pre-biopsy nomogram based on clinical variables and dNLR predicting absence of GG ≥ 2.; Figure S2: Calibration plots of the pre-biopsy nomogram based on clinical variables and PNI predicting absence of GG ≥ 2.; Table S1: Biomarker cut-offs with diagnostic estimates for GG ≥ 2 prediction; Table S2: Univariable analyses for GG ≥ 2 and any PCa detection in patients with PI-RADS ≥ 3 undergoing MRI-targeted and systematic biopsy.
