# Supplementary material for: Evaluation of the Predictive Role of Blood-Based Biomarkers in the Context of Suspicious Prostate MRI in Patients Undergoing Prostate Biopsy
Source: J Pers Med. 2021 Nov 19;11(11):1231. doi: 10.3390/jpm11111231 (PMC8625876; doi:10.3390/jpm11111231)
Supplement: Supplementary file 1 [file jpm-11-01231-s001.zip › jpm-1432987-supplementary.pdf]

Supplementary Materials

Supplementary Table S1. Biomarker cut-offs with diagnostic estimates for GG ≥ 2 prediction.

	Cut-off	Sens (%)	Spec (%)	NPV (%)	PPV (%)
NLR	≥2.75	35	82	48	72
dNLR	≥2.06	28	91	38	80
PLR	≥133.5	44	64	46	63
LMR	<2.07	17	93	45	78
SII	≥272.6	28	81	45	67
PNI	<52.8	48	79	52	76
De Ritis ratio	≥1.11	35	78	47	68

Abbreviations: dNLR: derived neutrophil-lymphocyte ratio; LMR: lymphocyte-monocyte ratio; NLR: neutrophil-lymphocyte ratio; NPV, negative predictive value; PLR, platelet-lymphocyte ratio; PNI: prognostic nutrition index; PPV, positive predictive value; Sens, sensitivity; Spec, specificity.

Supplementary Table S2. Univariable analyses for GG ≥ 2 and any PCa detection in patients with PI-RADS ≥ 3 undergoing MRI targeted and systematic biopsy.

	Univariable Analysis for GG ≥ 2 Detection			Univariable Analysis for PCA Detection		
Variable	OR	95% CI	*p*-value	OR	95% CI	*p*-value
Age (per 1 year)	1.07	1.04–1.10	<0.001	1.08	1.05–1.11	<0.001
PSA (per 1 unit)	1.05	1.01–1.08	0.009	1.04	1.00–1.07	0.037
PSAD (per 0.1 unit)	1.80	1.45–2.22	<0.001	1.81	1.42–2.30	<0.001
DRE (cT≥2)	4.87	2.64–8.98	<0.001	4.69	2.31–9.54	<0.001
PIRADS 3	Ref.	Ref.		Ref.	Ref.	
PI-RADS 4	7.04	2.62–18.95	<0.001	5.46	2.42–12.32	<0.001
PI-RADS 5	24.06	8.50–68.12	<0.001	25.37	9.85–65.36	<0.001
NLR (high vs. low)	2.39	1.41–4.05	0.001	2.45	1.36–4.42	0.003
dNLR (high vs. low)	3.67	1.91–7.07	<0.001	4.06	1.86–8.87	<0.001
PLR (high vs. low)	1.42	0.88–2.29	0.154	1.57	0.93–2.66	0.09
LMR (high vs. low)	0.35	0.16–0.75	0.007	0.21	0.07–0.60	0.004
SII (high vs. low)	1.69	0.99–2.87	0.055	1.61	0.90–2.87	0.109
De Ritis ratio (high vs. low)	1.62	0.99–2.63	0.053	1.70	1.00–2.88	0.051
mGPS (per 1 unit)	0.94	0.48–1.87	0.868	1.37	0.63–3.00	0.43
PNI (high vs. low)	0.39	0.24–0.65	<0.001	0.32	0.18–0.56	<0.001

Abbreviations: dNLR: derived neutrophil-lymphocyte ratio; DRE: digital rectal examination; GG: Gleason Grade; LMR: lymphocyte-monocyte ratio; *n*: number; mGPS: modified Glasgow prognostic score; NLR: neutrophil-lymphocyte ratio; PCA: prostate cancer; PI-RADS: Prostate Imaging-Reporting and Data System; PLR, platelet-lymphocyte ratio; PNI: prognostic nutrition index; PSA: prostate-specific antigen; PSAD: PSA density; SII: systemic immune-inflammation index.

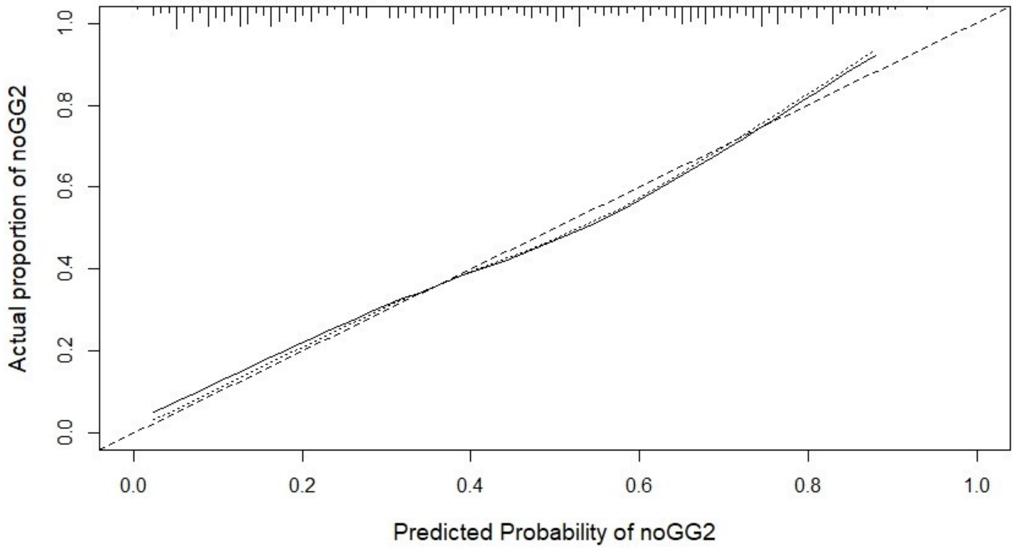

Supplementary Figure S1. Calibration plots of the pre-biopsy nomogram based on clinical variables and dNLR predicting absence of GG ≥ 2.

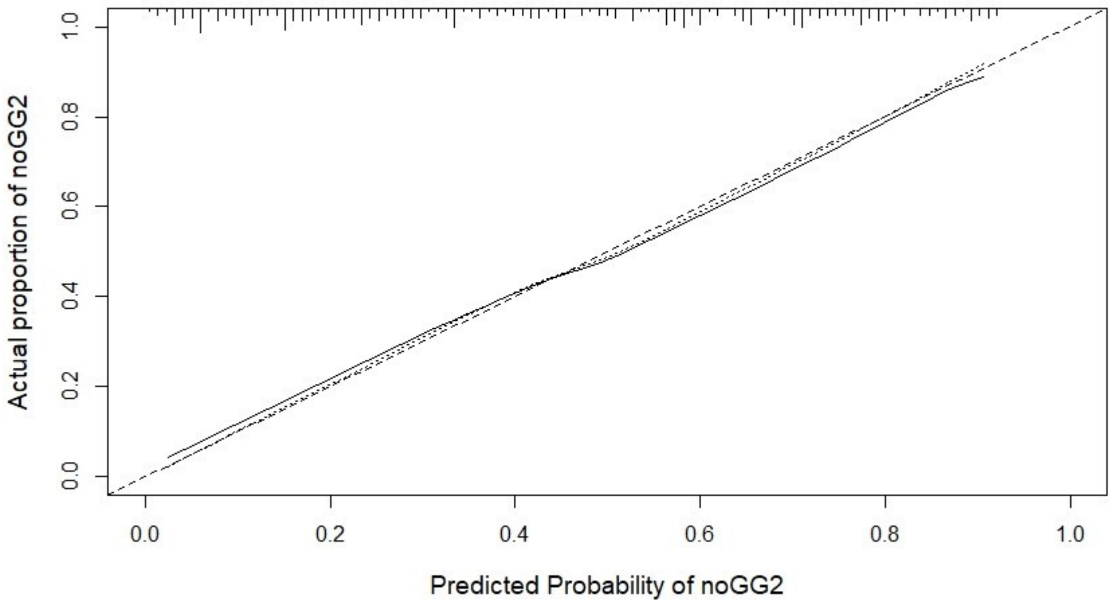

Supplementary Figure S2. Calibration plots of the pre-biopsy nomogram based on clinical variables and PNI predicting absence of GG ≥ 2.
